# Switchgrass Cultivar/Ecotype Selection and Management for Biofuels in the Upper Southeast USA

**DOI:** 10.1155/2014/937594

**Published:** 2014-06-29

**Authors:** Rocky Lemus, David J. Parrish, Dale D. Wolf

**Affiliations:** ^1^Department of Plant and Soil Sciences, Mississippi State University, Starkville, MS 39762, USA; ^2^Department of Crop and Soil Environmental Sciences, Virginia Tech, Blacksburg, VA 24061, USA

## Abstract

Switchgrass (*Panicum virgatum* L.), a perennial warm-season grass indigenous to the eastern USA, has potential as a biofuels feedstock. The objective of this study was to investigate the performance of upland and lowland switchgrass cultivars under different environments and management treatments. Four cultivars of switchgrass were evaluated from 2000 to 2001 under two management regimes in plots established in 1992 at eight locations in the upper southeastern USA. Two management treatments included 1) a single annual harvest (in late October to early November) and a single application of 50 kg N/ha/yr and 2) two annual harvests (in midsummer and November) and a split application of 100 kg N/ha/yr. Biomass yields averaged 15 Mg/ha/yr and ranged from 10 to 22 Mg/ha/yr across cultivars, managements, locations, and years. There was no yield advantage in taking two harvests of the lowland cultivars (Alamo and Kanlow). When harvested twice, upland cultivars (Cave-in-Rock and Shelter) provided yields equivalent to the lowland ecotypes. Tiller density was 36% lower in stands cutting only once per year, but the stands appeared vigorous after nine years of such management. Lowland cultivars and a one-cutting management (after the tops have senesced) using low rates of applied N (50 kg/ha) are recommended.

## 1. Introduction

Herbaceous biomass is being investigated in the search for renewable energy sources. One frequently cited potential energy crop is switchgrass, a perennial, C_4_ grass, with 19 MJ/kg energy content [1], and indigenous to the eastern two-thirds of the USA. It has been studied for production of biofuels (ethanol and diesel) and biopower (electricity and heat). At present, cofiring with coal appears to offer the most realistic potential use of switchgrass for bioenergy [2, 3]. The conversion of switchgrass to ethanol is a complicated process requiring a biorefinery. Small scale biorefineries are implemented with different enzymatic processes, but there has not been a process that has been proven reliable at this point. The major problems still exist in the saccharification process where cellulose and hemicellulose need to be broken down into sugars [4].

Bioenergy production systems using switchgrass can seek to maximize economic yields through proper cultivar choice, good harvest management (frequency, timing, and height of cutting), and optimization of fertilizer inputs (matching additions to crop needs). Currently there is limited information on the adaptation and management of different switchgrass ecotypes and cultivars in the upper southeast USA. Recent reports by Fike et al. [5, 6] have provided summations of a 10-year study in that region. This paper will look more closely at the final 2 years of that study and provide additional findings related to managing switchgrass for biomass.

One factor determining cultivar adaptation to a specific region is the origin of the genotype [7, 8]. Switchgrass cultivars and accessions all belong to one of two ecotypes or forms, based on their cytological and morphological characteristics [7]. Lowland ecotypes are tetraploid, taller, and thicker-stemmed and tend to be more of a bunch grass because of less vigorous rhizomes. Upland ecotypes are hexaploid or octaploid, shorter, and thinner-stemmed, with more vigorous rhizomes, and are perhaps more drought tolerant [7, 9]. Casler et al. [10] reported that yields of cultivars grown in the USA were correlated with the origin of the ecotype, with lowland ecotypes having higher yield potential.

Because the market price for biomass that will be used for energy purposes is likely to be lower than for many other commodities, there will be great value in optimizing switchgrass production economically, that is, achieving high yields while minimizing inputs [11]. One possible avenue to greater economic and agronomic sustainability is harvest management [7, 12]. Frequency and timing of biomass harvests can affect the supply of feedstock to processors, which can in turn affect market value. A harvest pattern that maintains energy and N reserves in roots can also encourage long-term productivity [7, 11, 13].

A single annual cutting in the late fall or winter has been considered practical for biomass production [11, 14], but harvesting more than once a year could be more economic, potentially increasing production and reducing storage requirements. However, more frequent harvests should be considered carefully because reduction of switchgrass stands has often been associated with too frequent harvests [7]. Cuomo et al. [13] estimated that three annual harvests of switchgrass decreased yield from 34 to 60% the following year in Nebraska. Switchgrass stand reductions from 39 to 58% were reported with two or three annual harvests as compared to one annual harvest in the Midwest [15, 16]. Similarly, multiple harvests per season reduced Alamo switchgrass yields in Texas [14]. In Oklahoma, switchgrass stands were severely reduced when cutting two or four times each season instead of once, and stand losses were lower when 10 cm of stubble was left instead of 5 cm [17].

Delaying switchgrass harvest until after frost allows translocation of materials from the aboveground biomass to the roots [7, 18, 19]. Parrish and Wolf [20] reported that this remobilization and translocation can cause a yield reduction in aboveground biomass of 10% or more between early September and late October. Sanderson et al. [12] have also observed that switchgrass yields decrease if the fall harvest is delayed from September to November. While these studies suggest that a single harvest is appropriate, two harvests per season have yielded more biomass in other studies [7, 11]. The differing results appear to relate to cultivar, timing, location, and perhaps other factors.

The profitability of switchgrass as a bioenergy crop might be enhanced through location-specific cultivar selection and harvest management. Information is needed to make informed recommendations for optimal switchgrass biofuel cropping systems in the upper southeast USA. The objective of this study was to investigate performance of upland and lowland switchgrass cultivars under different environments in this region and under two different management strategies.

## 2. Materials and Methods

The study was conducted during 2000 and 2001 as part of a larger study [2, 3], using switchgrass stands planted in 1992 at eight different sites across five states in the upper southeast USA (Table 1). Experimental plots were 6.1 × 2.4 m at all locations except at the Blacksburg site B, where plots were 6.1 m × 2.1 m. The cultivars utilized in the study included lowland (Alamo and Kanlow) and upland (Cave-in-Rock (CIR) and Shelter) ecotypes (Table 2). Two management treatments included (1) a single annual harvest (in late October to early November) and a single application of 50 kg N/ha/yr (low input and low management) and (2) two annual harvests (in midsummer and November) and a split application of 100 kg N/ha/yr (high input and intense management). All fall harvests were taken after aboveground biomass was highly senesced or dead. The first harvest in the two-cutting system was taken in late June or early July, when the upland cultivars were in an early head emergence stage and the lowland cultivars were at jointing or early boot stage. A 1 m wide strip was harvested from the center of each plot at a height of 10 cm using a flail type forage harvester (Carter Manufacturing Co., Brookston, IN) equipped with an electronic scale. Biomass subsamples were collected for dry matter determination. After each harvest, all border materials in each plot were mowed to 10 cm and residue was removed.

Nitrogen fertilizer was applied as ammonium nitrate (34-0-0). For the one-cutting management, 50 kg N/ha was applied in late May. The two-cutting management received a split application, with 50 kg N/ha applied in May and 50 kg N/ha applied after the first cutting. Phosphorus and potassium were applied as needed to maintain a medium level of fertility based on soil testing recommendations from each geographic location.

Stand densities were determined after the November 2001 harvest by visually evaluating stubble density. The visual ratings were made by a single individual across all locations to provide consistency. Plots were visually ranked from 0 = no stubble to 10 = a dense stubble (very little soil surface visible).

### 2.1. Statistical Analyses

The experimental design at each location was a split-plot design replicated four times, with the main plots being management and subplots being cultivars. Cultivars were randomized within management schemes. Cultivars, management schemes, and locations were considered to be fixed effects; years and reps were considered random. The location ∗ cultivar ∗ management ∗ year interaction was highly significant for biomass yields (Table 3); therefore, data were analyzed separately for each location. Analysis of variance procedures was conducted using the PROC MIXED procedure of SAS [21] at *α* = 0.05 to test the main effects and their interactions. Means were separated by the least significant difference (LSD) at *α* = 0.05. A site yield variation index, for cultivars in each management scheme, was calculated as the cultivar mean at each location minus the location grand mean [22].

## 3. Results and Discussion

### 3.1. Climatic Data

Essentially all switchgrass top growth occurs between April and September, so we have looked most closely at temperature and rainfall data during that interval. Rainfall patterns during this period varied with year and across locations. Two locations (KY and TN_2_) experienced slightly below normal rainfalls in both years, while rainfalls somewhat above 30-year averages were seen at several locations (Table 4). Overall, the April-to-September weather data in both years reflected little deviation from long-term means for precipitation and temperature. The NC site had the highest precipitation in both years, especially in 2000, as a result of a hurricane. Mean temperatures in 2001 were cooler than in 2000, particularly at VA_3_ and KY locations, but both years were above long-term averages overall, most notably in NC.

### 3.2. Cultivar and Management Differences across Years and Sites

Analysis revealed a significant management ∗ year ∗ cultivar interaction (Table 5). Due to the three-way interaction, data analysis was divided into main effects and two-way interactions for discussion purposes. Over the 2 years of this study, yields from the higher-intensity management (averaging 15.3 Mg/ha/yr across sites and cultivars) provided a 12% mean yield advantage over the lower-intensity system (13.5 Mg/ha/yr) (Table 5). Most of the observed yield advantages occurred in 2000, when two cuttings and 100 kg N/ha resulted in a 21% yield increase. The higher-intensity management was not consistently superior. In fact, it produced significantly higher yields across years and cultivars at only three sites (NC, TN_1_, and VA_1_), and it was outyielded by the lower-input management at the WV site.

Mean yields across managements, cultivars, and years ranged from 12.6 Mg/ha in NC to 19.5 Mg/ha in VA_2_. Interestingly, the yields of VA_2_ contrasted dramatically with those at VA_1_, located approximately 100 m east of VA_2_. The physical differences between these two sites included soil type, soil pH (at the end of the study), slope, and aspect but not temperature (except microenvironmentally) or rainfall. Although there was no yield correlation with growing season rainfall (April through September), there is the possibility that wetter, warmer conditions at NC and drier conditions at TN_1_ might have affected overall performance in these lower-yielding locations.

When looking at performance of the cultivars used in this study, Alamo and Kanlow were generally higher yielding than CIR and Shelter across locations, years, and managements. Shelter was the least productive or tended to be the least productive cultivar at all locations, having a 30% lower mean yield than Alamo. All cultivars generally had lower yields in TN_1_ and NC.

Management intensity had differential effects on cultivar performance (Table 6). No yield advantage was observed for Alamo and Kanlow under the higher-input management. In fact, their yields were significantly reduced when cutting twice at several locations, with the highest reductions at TN_2_ (~16%) and WV (~24%). Our data agree with others' findings, in which Alamo yields decreased with increasing harvest frequency [5, 6, 14]. In contrast to Alamo's and Kanlow's yield reductions with increased harvests, CIR and Shelter seasonal yields were increased by approximately 30% when cutting twice, making CIR yields equivalent to lowland cultivars. These data suggest that switchgrass management and perhaps especially harvest frequency should be both cultivar- and location-specific depending on soil type, climate, and fertility.

Under the two-cutting management, more biomass was typically removed in the June harvests than in November (Table 7), but there clearly were cultivar and location differences. When averaged across cultivars, no yield differences between harvests were observed at the three sites in VA; that is, each harvest was about 50% of the seasonal total. For all of the other sites except KY, only about one-third of the seasonal yield came in the November harvests.

When looking at seasonal distribution of biomass production by cultivar, CIR and Shelter had about 60% of their total yield removed in June under the two-cutting management when averaged across locations and years. On the other hand, Alamo and Kanlow had 5 to 10% less removed in June. These data indicate that, by midsummer in the upper Southeast USA, CIR and Shelter have produced more than half of their seasonal yield. Most of the first harvests occurred in the late boot (*R*_0_) to early seed head emergence (*R*_1_) stage [23]. Since CIR and Shelter developed seed heads earlier, the June harvest date could have given some advantage to the lowland cultivars due to their extended vegetative growth. Also, relative to some forage species, switchgrass has a slow recovery time for regrowth, which might be different for each cultivar, affecting yield sustainability. Energy resources that might have been dedicated to increase total biomass yield will have to be expended for regrowth.

### 3.3. Ecotype Differences in Response to Management

Our results indicate that the upland ecotypes (CIR and Shelter) responded differently from the lowland ecotypes (Alamo and Kanlow) to management treatments. Therefore, we have made the assumption that these cultivars are representative of their respective ecotypes and pooled data by ecotype (Table 8). The general pattern observed was that the upland ecotypes produced greater yields (14.9 versus 11.4 Mg/ha) with higher inputs, while the lowland ecotypes did not benefit from the more intense management. At VA_1_ and TN_1_, the lowland ecotype responded atypically, showing a yield increase with two cuttings and more N. In contrast, yield reductions were observed with the higher inputs in TN_2_ (23%) and WV (21%). The upland ecotype increased its yields with higher inputs at all locations except WV. Overall higher-input management of upland types produced about 31% more biomass than the lower inputs. The yield response of the upland ecotype to higher inputs ranged from nonsignificant in WV to 65% in NC.

One of the reasons for the generally better performance of lowland cultivars (with or without higher inputs) can be related to their phenological response to day length. Alamo and Kanlow remained vegetative (did not produce visible panicles) until later in the season and this prolonged vegetative growth might be expected to result in yield increases [9, 11].

### 3.4. Visual Stand Evaluation

Stand densities were visually rated after the November 2001 harvest, 10 years into the larger study [5, 6]. Management played a major role in stand density. Plots receiving the intense management had denser stubble, that is, more tillers (Table 9). Plots that received one cutting and 50 kg N/ha/yr had a 36% lower stand density rating as determined by stubble. The lowest densities observed (at VA_2_) were produced under the one-cutting, low-N management. In fact, stands for the lower-input management were generally less dense for all locations in VA for both ecotypes, but yields were not reduced: VA_2_ and VA_3_ were among the highest yielding locations (Table 5). The increase in stand density with the higher-intensity management was perhaps due to stimulation of tiller development by the greater amount of N applied, but it could also have been due to tillering triggered by the June cutting. The midsummer cutting at 10 cm may have caused axillary buds on the culms to become active and produce additional branching, if not true tillers.

### 3.5. Cultivar Origins, Adaptation, and Yield Stability

**Figure 1:**
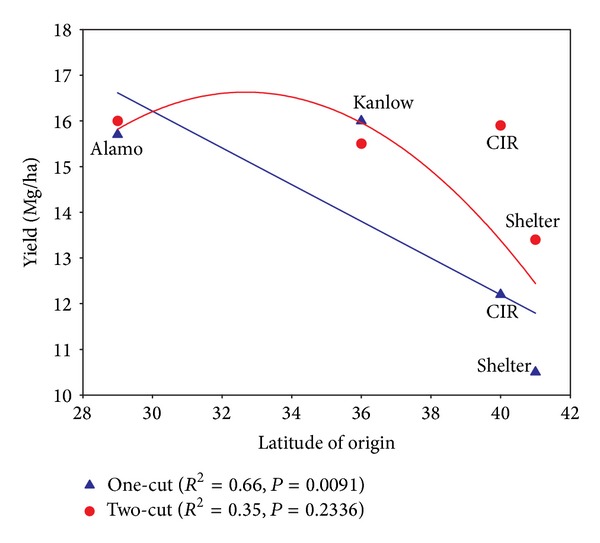
Relationship between latitude of origin and yield of four switchgrass cultivars grown under two management systems in the upper southeast USA. The one-cut management included 50 kg N ha/yr, and the two-cut management involved applying 100 kg N/ha/yr. Yield data are the means for each cultivar averaged across sites and years from Table 4.

Cultivar ∗ environment interactions must be examined carefully when trying to determine adaptation of a cultivar to a specific area. When comparing multiple cultivars over a series of locations and years, their relative rankings often change, making it difficult or impossible to identify a “best” cultivar. Because switchgrass cultivars have generally been derived from naturally occurring populations of the species at a particular locale, we might expect various cultivars' adaptation and yield at a new location to vary with their point of origin (latitude and/or longitude). This concept has been examined most closely by Casler and colleagues [10, 24–26]. To examine the relationship between yield and a cultivar's provenance, the mean biomass yields for each cultivar were plotted against its latitude of origin (Figure 1).

Figure 1 suggests that cultivars of southern origin might be more productive at the latitudes of this study. That conclusion is mitigated by the very small number of cultivars examined, but Casler and colleagues have made the point much more amply [10, 24–26]. Moving switchgrass cultivars up to 500 km [27] or one USDA hardiness zone [10] north of their point of origin can result in significant yield increases, presumably because of a prolonged period of vegetative growth (and the observation that biomass production tends to cease shortly after anthesis) [11].

Figure 1 also suggests that the effect of provenance on yield is most evident under lower-input management. With two cuttings and 100 kg N/ha/yr, the four cultivars looked much more alike in their yield potential and/or stability. There was still some tendency for the cultivars of more southern origin to be higher yielding, but the effect was much less magnified.

If we look at these data in the context of ecotype, the lowland cultivars (Alamo and Kanlow) performed better at all locations. The reason for the poorer performance of the upland cultivars may be related to their more northern origin and consequent responses to photoperiod. The superior yields of the lowland cultivars may have been a function of their inherent morphological characteristics (taller, coarser stems, and a more bunch-type habit) or it may have been a result of later flowering and extended duration of vegetative growth, which would have resulted from moving them northward [28].

**Figure 2:**
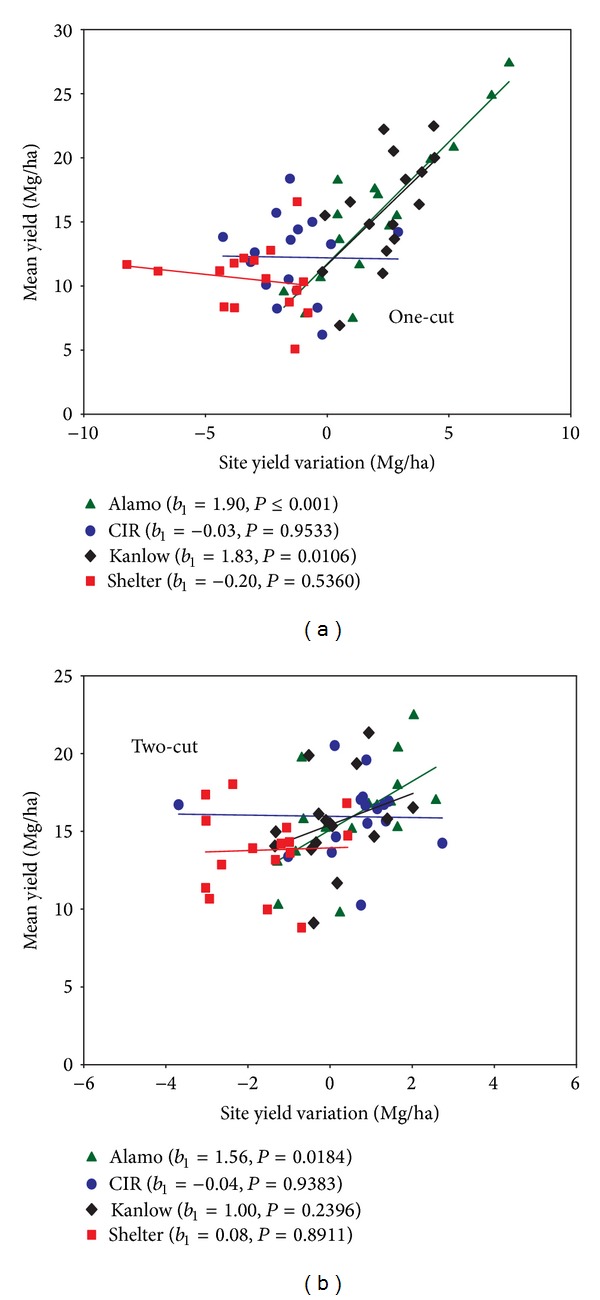
Regression of each switchgrass cultivar's mean yield for each location and year against its site yield variation for (a) lower-input, one-cut or (b) higher-input, two-cut systems. A cultivar's site yield variation was calculated as its mean yield at a location minus the location's grand mean yield [19].

Indexing the yield means of cultivars across sites [22] revealed that Alamo and Kanlow were less stable across locations [Slope  (*b*_1_) ≥ 1] than CIR and Shelter were, under both cutting managements (Figure 2). Put in another way, there was greater sensitivity to site with the lowland cultivars. They did better than the average in yield but their yields were more sensitive to location. Cave-in-Rock behaved similarly at all locations under either management system, while Shelter varied by location in its response to the higher-input system. The data suggest that the upper southeast USA can certainly employ lowland switchgrass cultivars, but site-specific studies may be needed to optimize cultivar choice. Stout [29] has shown that soil physical and chemical properties can affect switchgrass productivity in the northeastern USA. The dramatic differences between two closely located sites in VA (VA_1_ and VA_2_) certainly bear out the notion that soil- or site-specific factors may need to be taken into consideration when utilizing up- or lowland cultivars.

## 4. Conclusions

Switchgrass demonstrated high productivity across a wide geographic region in the upper southeast USA. Alamo and Kanlow (lowland cultivars) produced higher yields than CIR and Shelter (upland cultivars). Differences in yield among cultivars were mainly related to management and ecotype. Results indicate that both switchgrass ecotypes can be successfully harvested once or twice per year in the upper southeast. For the upland ecotype, a two-cutting system can increase yields without affecting stand persistence. For the lowlands, a single cutting at the end of the growing season will provide as much or more yield than multiple cuttings. Yield differences among cultivars at each location suggest that cultivar choice and management may need to be site-specific.

Incorporating switchgrass into an energy system could lead to major improvements in the sustainability of agroecosystems. Developing harvest management practices that maximize yield, with the least impact on environmental quality and with the greatest economic return, will require a greater understanding of the interaction between the environment, ecotypes, and soil dynamics. Further testing of these and other cultivars under a combination of cutting frequencies and N fertilization rates should be performed in the upper southeast. These management strategies will determine how these variables may affect switchgrass development and persistence. Results of these studies could also be used to develop an index of selection for cultivars and sites that will optimize biofuels production and help identify new cultivars or lines more adapted to the upper southeast. This will also aid in breeding programs and future screening of cultivars with specific biofuel qualities such as higher yields and lower lignin, sulfur, and NO_*x*_ content.

## Figures and Tables

**Table 1:** Location, elevation, soil series, pH, and hardiness zones of sites where switchgrass varieties were evaluated as a biofuels crop in 2000 and 2001. The pH values collected in 1992 during the establishment of the switchgrass plots at all locations.

State	Town	Lat. N	Long. W	Elev.	Soil series	pH	Hardiness Zone [30]
		Degree, Min		m		
KY	Princeton	37°06′	87°49′	173	Tilsit, 2 to 6% slope (fine-silty, mixed, mesic Typic fragiudults)	6.8	7a

NC	Raleigh	35°43′	78°40′	120	Cecil, 5 to 10% slope (fine, kaolinitic thermic Typic Kanhapludults)	6.3	7b

TN	Jackson (TN_1_)	35°37′	88°55′	120	Deanburg, 2 to 5% slope (fine-loamy, mixed, thermic Ultic Hapludalfs)	5.7	8a
	Knoxville (TN_2_)	35°53′	83°57′	250	Etowah, 5 to 12% slope (fine-loamy, siliceous, thermic Typic Paleudults)	5.5	7a

VA	Blacksburg	37°11′	80°25′	600			
	Site A (VA_1_)				Chatter, 2 to 5% slope (clayey, kaolinitic, mesic Typic Paleudult)	6.2	6b
	Site B (VA_2_)				Shot tower, 10 to 15% (clayey, kaolinitic, mesic Typic Paleudults)	6.2	6b
	Orange (VA_3_)	38°13′	78°07′	156	Davidson, 10% slope (clayey, kaolinitic, thermic Rhodic Kandiudults)	5.6	7a

WV	Morgantown	39°37′	79°55′	378	Dormont, 2 to 5% slope (fine-loamy, mixed, mesic Ultic Hapludalfs)	6.2	6b

**Table 2:** Description of switchgrass cultivars evaluated at eight locations in the upper southeast USA in 2000 and 2001.

Cultivar	Ploidy			Released [31]	
	Ecotype [32]	Level [33]	Origin [31]	Year	By
Cave-in-Rock	Upland	Octaploid	Southern Illinois	1973	NRCS PMC∗, Elsberry, MO
Shelter	Upland	Octaploid	St. Mary's, WV	1986	NRCS PMC, Big Flats, NY
Alamo	Lowland	Tetraploid	Southern Texas	1978	NRCS PMC, Knox City, TX
Kanlow	Lowland	Tetraploid	Central Oklahoma	1963	NRCS PMC, Manhattan, KS

*NRCS PMC: Natural Resources Conservation Service, Plant Materials Center.

**Table 3:** Statistical significance of fixed and random effects on biomass yield for four switchgrass cultivars and two cutting managements across eight locations in 2001 and 2002 in the upper southeast USA.

Source of variation	df	Mean square	Significant of *F* ratio
Year (Yr)	1	13.3	NS
Location (Loc)	7	434.3	∗
Yr∗Loc	7	123.6	∗
Management (Mgt)	1	363.1	∗
Yr∗Mgt	1	150.1	∗
Loc∗Mgt	7	77.6	∗
Yr∗Loc∗Mgt	7	29.5	∗
Cultivar (Cv)	3	387.6	∗
Yr∗Cv	3	5.1	NS
Loc∗Cv	21	25.6	∗
Yr∗Loc∗Cv	21	11.9	∗
Mgt∗Cv	3	143.7	∗
Yr∗Mgt∗Cv	3	5.6	NS
Loc∗Mgt∗Cv	21	11.7	∗
Yr∗Loc∗Mgt∗Cv	21	9.7	∗

NS: not significant.

∗Significant at the 0.05 level.

**Table 4:** Observed and long-term means for rainfall and temperature data during April through September (A–S) near sites on which switchgrass was grown in the upper Southeast USA. Departure from long-term (30 yr) normal was obtained by subtracting total precipitation and mean temperature from their respective long-term values.

Location^1^	Long-term	A–S observed		Departure from long-term	
	A–S	2000	2001	2000	2001
	Precipitation (cm)				
KY	64.3	62.1	62.2	−2.2	−2.1
NC	58.6	101.3	78.3	42.7	19.7
TN_1_	57.9	74.0	63.3	16.1	5.4
TN_2_	65.6	54.2	60.5	−11.4	−5.1
VA_1,2_	51.6	66.2	61.9	14.6	10.3
VA_3_	58.7	62.3	53.4	3.6	−5.3
WV	58.5	62.6	62.0	4.1	3.5
Mean	**59.3**	**69.0**	**63.1**	**9.6**	**3.8**

	Mean temperature (°C)				
KY	21.6	25.7	22.3	4.2	0.8
NC	18.7	24.2	21.6	5.5	2.9
TN_1_	21.0	23.8	21.2	2.9	0.2
TN_2_	22.1	25.6	22.7	3.5	0.5
VA_1,2_	17.8	20.4	18.0	2.6	0.2
VA_3_	20.0	22.4	19.9	2.4	−0.1
WV	18.5	20.4	18.3	1.9	−0.2
Mean	**19.9**	**23.2**	**20.6**	**3.3**	**0.6**

^1^Locations: KY (Princeton), NC (Raleigh), TN_1_ (Jackson), TN_2_ (Knoxville), VA_1,2 _(Blacksburg), VA_3_ (Orange), and WV (Morgantown).

**Table 5:** Effect of management (one-cut (low input and management) and two-cut (high input and management)), year, and cultivar on yield of switchgrass grown at eight locations in upper southeast USA (CIR: Cave-in-Rock).

Cuttings/N	Year	Cultivar	Location^1^								Mean	LSD_0.05_^3^
			KY	NC	TN_1_	TN_2_	VA_1_	VA_2_	VA_3_	WV		
			Yield (Mg/ha)									
One/50 kg	2000	Alamo	15.5	14.7	7.8	20.8	9.5	17.1	19.8	15.5	15.1	4.5
		Kanlow	16.4	14.8	11.0	15.5	11.1	18.9	16.6	18.3	15.3	3.7
		CIR	10.1	10.5	8.3	14.4	14.2	11.9	15.0	13.6	12.2	3.1
		Shelter	8.4	8.3	7.9	11.8	10.3	12.0	11.2	12.8	10.3	3.0
		Mean	12.6	12.1	8.7	15.6	11.3	15.0	15.6	15.1	13.2	2.0
		LSD_0.05_^2^	2.3	3.7	2.8	3.1	4.2	4.9	5.2	3.4	1.4	—
	2001	Alamo	13.6	7.4	11.6	24.9	10.6	27.4	17.6	18.2	16.4	4.9
		Kanlow	14.8	6.9	12.7	22.4	13.7	22.2	20.0	20.5	16.8	5.3
		CIR	13.2	6.2	8.2	13.8	9.6	18.4	12.6	15.7	12.2	3.6
		Shelter	10.6	5.1	8.7	11.2	9.7	11.7	12.2	16.7	10.7	3.4
		Mean	13.1	6.4	10.3	18.1	10.9	19.9	15.6	17.8	14.0	2.4
		LSD_0.05_^2^	3.1	1.8	3.0	4.7	3.6	8.0	6.4	3.2	1.7	—
		Mean	**12.8**	**9.2**	**9.5**	**16.9**	**11.1**	**17.4**	**15.6**	**16.4**	**13.6**	**2.1**
Two/100 kg	2000	Alamo	17.0	16.7	10.2	16.9	15.8	22.4	20.4	15.2	16.8	4.6
		Kanlow	15.8	15.7	11.7	14.1	16.1	21.3	19.4	15.4	16.2	3.7
		CIR	13.4	16.7	14.2	16.7	17.2	20.5	19.6	16.4	16.8	3.3
		Shelter	11.4	13.9	9.8	14.2	16.8	17.4	15.7	14.3	14.2	3.6
		Mean	14.4	15.7	11.5	15.5	16.5	20.4	18.7	15.3	16.0	1.8
		LSD_0.05_^2^	2.5	1.9	NS^5^	1.9	NS	3.2	NS	0.8	1.2	—
	2001	Alamo	15.2	9.7	16.6	17.9	13.7	19.7	15.1	13.0	15.1	4.6
		Kanlow	14.7	9.1	15.5	15.0	16.5	19.9	14.2	13.8	14.8	4.4
		CIR	13.6	10.2	16.9	17.0	14.6	16.7	15.5	15.7	15.1	3.4
		Shelter	10.7	8.8	12.9	15.2	13.2	18.0	13.6	14.7	13.4	3.0
		Mean	13.6	9.5	15.5	16.3	14.5	18.6	14.6	14.3	14.6	1.9
		LSD_0.05_^2^	1.7	NS	NS	2.4	3.2	NS	NS	1.4	1.3	—
		Mean	**14.0**	**12.6**	**13.5**	**15.9**	**15.5**	**19.5**	**16.7**	**14.8**	**15.3**	**0.9**

		LSD_0.05_^4^	NS	1.9	1.7	NS	1.2	NS	NS	1.2	1.3	—

^1^Locations: KY (Princeton), NC (Raleigh), TN_1_ (Jackson), TN_2_ (Knoxville), VA_1,2_ (Blacksburg), VA_3_ (Orange), and WV (Morgantown); ^2^LSD for comparison of cultivars within cutting, year, and location; ^3^LSD for comparison of locations within a management, year, and cultivar; ^4^LSD for comparison of means between managements; ^5^NS: not significant.

**Table 6:** Effect of management (one-cut (low input and management) and two-cut (high input and management)) and cultivar on yield of switchgrass grown at eight locations in upper southeast USA. Data are averaged across 2 years (CIR: Cave-in-Rock).

Cuttings/N	Cultivar	Location^1^								Mean	LSD_0.05_^4^
		KY	NC	TN_1_	TN_2_	VA_1_	VA_2_	VA_3_	WV		
		Yield (Mg/ha)									
One/50 kg	Alamo	14.5	11.0	9.7	22.8	10.1	22.2	18.7	16.9	15.7	4.1
	Kanlow	15.6	10.9	11.9	19.0	12.4	20.6	18.3	19.4	16.0	3.7
	CIR	11.7	8.3	8.3	14.1	11.9	15.1	13.8	14.7	12.2	2.9
	Shelter	9.5	6.6	8.3	11.5	10.0	11.8	11.7	14.7	10.5	2.3
	Mean	**12.8**	**9.2**	**9.6**	**16.9**	**11.1**	**17.4**	**15.6**	**16.4**	**13.6**	**2.4**
	LSD_0.05_^2^	2.1	3.7	2.1	3.3	2.8	5.3	3.8	2.5	1.6	—
Two/100 kg	Alamo	16.1	13.2	13.4	17.4	14.7	21.1	17.7	14.1	16.0	3.7
	CIR	13.5	13.4	15.6	16.8	15.9	18.6	17.5	16.0	15.9	2.8
	Kanlow	15.2	12.4	13.6	14.5	16.3	20.6	16.8	14.6	15.5	3.1
	Shelter	11.0	11.3	11.4	14.7	15.0	17.7	14.6	14.5	13.8	2.5
	Mean	**14.0**	**12.6**	**13.5**	**15.9**	**15.5**	**19.5**	**16.7**	**14.8**	**15.3**	**0.9**
	LSD_0.05_^2^	1.4	NS^5^	4.1	NS	2.6	NS	2.1	1.0	1.3	—

	LSD_0.05_^3^	0.5	1.3	NS	NS	3.5	NS	NS	1.5	1.3	—

^1^Locations: KY (Princeton), NC (Raleigh), TN_1_ (Jackson), TN_2_ (Knoxville), VA_1,2 _(Blacksburg), VA_3_ (Orange), and WV (Morgantown).

^2^LSD for comparison of cultivars within a management and location.

^3^LSD for comparison of means within location.

^4^LSD for comparison of locations within each management and cultivar.

^5^NS: not significant.

**Table 7:** Effect of harvest date (first and second harvests) and cultivar on biomass yield of switchgrass grown at eight locations across upper southeast USA under two-cuts (high input and management). Data are averaged across 2 years (CIR: Cave-in-Rock).

Harvest	Cultivar	Location^1^								Mean	LSD_0.05_^3^
		KY	NC	TN_1_	TN_2_	VA_1_	VA_2_	VA_3_	WV		
		(Mg/ha)									
June (first)	Alamo	7.1	8.9	7.7	10.4	6.8	9.3	7.8	7.7	8.2	2.1
	Kanlow	8.3	8.7	9.0	9.8	8.5	10.3	9.1	9.4	9.1	2.0
	CIR	8.2	8.9	10.7	11.7	9.3	10.7	9.7	10.7	10.0	2.1
	Shelter	6.3	7.6	8.0	10.4	8.9	10.0	8.6	10.0	8.7	1.9
	Mean	**7.5**	**8.5**	**8.8**	**10.6**	**8.3**	**10.1**	**8.8**	**9.4**	**9.0**	**1.7**
	LSD_0.05_^2^	1.2	0.8	2.7	1.0	0.8	1.2	1.0	0.6	0.5	—
November (second)	Alamo	9.0	4.3	5.7	7.0	7.9	11.8	9.9	6.4	7.8	2.4
	Kanlow	6.9	3.7	4.6	4.7	7.8	10.3	7.7	5.2	6.4	2.4
	CIR	5.3	4.6	4.9	5.2	6.6	7.9	7.9	5.3	6.0	1.8
	Shelter	4.7	3.7	3.3	4.3	6.1	7.7	6.1	4.5	5.1	1.4
	Mean	**6.5**	**4.1**	**4.6**	**5.3**	**7.1**	**9.4**	**7.9**	**5.4**	**6.3**	**1.8**
	LSD_0.05_^2^	1.1	0.9	1.4	0.5	1.4	1.4	1.6	0.7	0.5	—

	LSD_0.05_^4^	0.4	1.4	1.2	1.0	NS^5^	NS	NS	1.3	0.4	—

^1^Locations: KY (Princeton), NC (Raleigh), TN_1_ (Jackson), TN_2_ (Knoxville), VA_1,2 _(Blacksburg), VA_3_ (Orange), and WV (Morgantown).

^2^LSD for comparison of cultivars within a harvest and location.

^3^LSD for comparison of locations within a harvest and cultivar.

^4^LSD for comparison of means within a location.

^5^NS: not significant.

**Table 8:** Effect of ecotype and management (one-cut (low input and management) and two-cut (high input and management)) on yield of switchgrass at eight locations across upper southeast USA. Data are averaged across cultivars (Alamo + Kanlow = lowland; Cave-in-Rock + Shelter = upland) and 2 years.

Ecotype	Cuttings/N	Location^1^								Mean	LSD_0.05_^3^
		KY	NC	TN_1_	TN_2_	VA_1_	VA_2_	VA_3_	WV		
		Yield (Mg/ha)									
Lowland	One/50	15.1	11.0	10.8	20.9	11.2	21.4	18.5	18.1	15.9	2.7
	Two/100	15.7	12.8	13.5	16.0	15.5	20.8	17.2	14.3	15.7	2.3
	Mean	**15.4**	**11.9**	**12.2**	**18.5**	**13.4**	**21.1**	**17.9**	**16.2**	**15.8**	**1.9**
	LSD_0.05_^2^	NS^5^	NS	2.6	2.7	1.9	NS	NS	1.6	NS	—
Upland	One/50	10.6	7.5	8.3	12.8	11.0	13.5	12.7	14.7	11.4	1.9
	Two/100	12.2	12.4	13.5	15.8	15.4	18.2	16.1	15.3	14.9	2.0
	Mean	**11.4**	**10.0**	**10.9**	**14.3**	**13.2**	**15.9**	**14.4**	**15.0**	**13.1**	**1.6**
	LSD_0.05_^2^	1.6	1.2	1.7	1.3	1.3	2.3	1.7	NS	0.7	—

	LSD_0.05_^4^	1.0	NS	NS	1.7	NS	2.2	2.3	1.2	0.8	—

^1^Locations: KY (Princeton), NC (Raleigh), TN_1_ (Jackson), TN_2_ (Knoxville), VA_1,2 _(Blacksburg), VA_3_ (Orange), and WV (Morgantown).

^2^LSD for comparison of managements within a location and ecotype.

^3^LSD for comparison of locations within an ecotype and management.

^4^LSD for comparison of ecotype means within a location.

^5^NS: not significant.

**Table 9:** Stand density ratings (0: least dense, 10: most dense) in November 2001 of four switchgrass cultivars grown under two managements at eight locations in upper southeast USA. Plots had been harvested once (in November) and provided 50 kg N/ha/yr or harvested twice (midsummer and November) and provided 100 kg N/ha/yr for 10 years (CIR: Cave-in-Rock).

Cuttings/N	Cultivar	Location^1^								Mean	LSD_0.05_^4^
		KY	NC	TN_1_	TN_2_	VA_1_	VA_2_	VA_3_	WV		
		Stand Rating (0 to 10)									
One/50 kg	Alamo	9.8	6.8	6.2	7.0	4.8	2.9	6.2	8.0	6.4	2.6
	Kanlow	10.0	8.2	8.2	7.5	6.8	3.5	6.6	9.5	7.5	1.9
	CIR	9.5	7.8	8.0	6.5	6.6	4.2	7.1	5.5	6.9	1.6
	Shelter	8.2	6.8	8.2	7.2	7.0	3.9	5.6	6.5	6.6	2.4
	Mean	**9.4**	**7.4**	**7.7**	**7.0**	**6.3**	**3.6**	**6.4**	**7.4**	**6.9**	**0.8**
	LSD_0.05_^2^	1.1	NS^5^	NS	NS	1.0	NS	NS	2.3	0.6	—
Two/100 kg	Alamo	10.0	9.5	8.8	9.2	9.8	8.6	8.9	10.0	9.4	0.9
	Kanlow	10.0	8.8	9.0	9.8	10.0	9.3	9.6	9.5	9.5	1.2
	CIR	10.0	9.5	9.2	9.5	9.6	8.6	9.2	8.5	9.2	1.0
	Shelter	10.0	9.2	9.5	9.8	9.8	9.1	9.1	9.8	9.5	0.7
	Mean	**10.0**	**9.2**	**9.1**	**9.6**	**9.8**	**8.9**	**9.2**	**9.4**	**9.4**	**0.4**
	LSD_0.05_^2^	NS	0.5	NS	NS	NS	NS	NS	0.8	NS	—

	LSD_0.05_^3^	0.4	0.7	1.1	1.3	0.4	0.6	0.9	0.9	0.3	—

^1^Locations: KY (Princeton), NC (Raleigh), TN_1_ (Jackson), TN_2_ (Knoxville), VA_1,2_ (Blacksburg), VA_3_ (Orange), and WV (Morgantown).

^2^LSD for comparison of cultivars within a management and location.

^3^LSD for comparison of managements within a location.

^4^LSD for comparison of locations within each management and cultivar.

^5^NS: not significant.
